# The Biodiversity of the Microbiota Producing Heat-Resistant Enzymes Responsible for Spoilage in Processed Bovine Milk and Dairy Products

**DOI:** 10.3389/fmicb.2017.00302

**Published:** 2017-03-01

**Authors:** Solimar G. Machado, François Baglinière, Sophie Marchand, Els Van Coillie, Maria C. D. Vanetti, Jan De Block, Marc Heyndrickx

**Affiliations:** ^1^Instituto Federal do Norte de Minas Gerais – Campus SalinasSalinas, Brazil; ^2^Department of Microbiology, Universidade Federal de ViçosaViçosa, Brazil; ^3^Technology and Food Science Unit, Flanders Research Institute for Agriculture, Fischeries and Food (ILVO)Melle, Belgium; ^4^Department of Pathology, Bacteriology and Poultry Diseases, Ghent UniversityMerelbeke, Belgium

**Keywords:** microbial dynamics, psychrotrophic, *Pseudomonas*, *Serratia*, peptidase, lipase, heat-resistant enzyme, spoilage

## Abstract

Raw bovine milk is highly nutritious as well as pH-neutral, providing the ideal conditions for microbial growth. The microbiota of raw milk is diverse and originates from several sources of contamination including the external udder surface, milking equipment, air, water, feed, grass, feces, and soil. Many bacterial and fungal species can be found in raw milk. The autochthonous microbiota of raw milk immediately after milking generally comprises lactic acid bacteria such as *Lactococcus*, *Lactobacillus*, *Streptococcus*, and *Leuconostoc* species, which are technologically important for the dairy industry, although they do occasionally cause spoilage of dairy products. Differences in milking practices and storage conditions on each continent, country and region result in variable microbial population structures in raw milk. Raw milk is usually stored at cold temperatures, e.g., about 4°C before processing to reduce the growth of most bacteria. However, psychrotrophic bacteria can proliferate and contribute to spoilage of ultra-high temperature (UHT) treated and sterilized milk and other dairy products with a long shelf life due to their ability to produce extracellular heat resistant enzymes such as peptidases and lipases. Worldwide, species of *Pseudomonas*, with the ability to produce these spoilage enzymes, are the most common contaminants isolated from cold raw milk although other genera such as *Serratia* are also reported as important milk spoilers, while for others more research is needed on the heat resistance of the spoilage enzymes produced. The residual activity of extracellular enzymes after high heat treatment may lead to technological problems (off flavors, physico-chemical instability) during the shelf life of milk and dairy products. This review covers the contamination patterns of cold raw milk in several parts of the world, the growth potential of psychrotrophic bacteria, their ability to produce extracellular heat-resistant enzymes and the consequences for dairy products with a long shelf life. This problem is of increasing importance because of the large worldwide trade in fluid milk and milk powder.

## Introduction

The dairy industry has a long tradition of safeguarding the safety and quality of consumer milk. Two main processes are at the basis of this quality system: cooling of the raw milk to temperatures below 7–10°C until processing and heating the milk in a dairy plant to produce different types of consumer milk depending on the heating process applied: pasteurized, extended shelf life (ESL), ultra-high temperature treated (UHT) or sterilized milk. The most consumed milk worldwide is either pasteurized or UHT. These heating processes eliminate pathogens and increase the shelf life of unopened packages. Pasteurized milk should be stored at refrigeration temperature (4–7°C) for a shelf life of about 2 weeks. On the other hand, UHT milk can be stored for 6–12 months at ambient temperature. However, spoilage can still happen during the predicted shelf life. Spoilage can be considered as any change, which renders a food product unacceptable for human consumption or for business to business trading. Besides physical damage to milk packaging, it is manifested by growth of microorganisms or enzymatic reactions leading to souring, changes in texture, or development of off-flavors.

The spoilage phenomena and mechanisms can be very different in the various types of consumer milk. The predicted and obtained shelf life of pasteurized milk is mainly determined by the presence and growth of aerobic psychrotrophic endospore formers of which members of the *Bacillus cereus* group are the most important spoilers, but other species of the genus *Bacillus* and allied genera are involved as well. Because the endospores resist the pasteurization process, the main spoilage mechanism is their subsequent germination and outgrowth with the production of spoilage enzymes in the pasteurized milk. Gopal et al. (2015) published a recent review for this type of spoilage. Nevertheless, it cannot be excluded that in some dairies post-pasteurization by mainly pseudomonads is still a problem leading to spoiled packages by the production of spoilage enzymes during psychrotrophic growth under refrigeration. Moreover, spoilage enzymes are already produced in the cooled raw milk by psychrotrophs like endospore formers in the vegetative state and pseudomonads. The heat resistance under pasteurization or the role of these enzymes in spoilage is largely unknown and probably of less importance in this type of milk. Native milk proteases and lipases may also be important factors limiting the shelf life of pasteurized milk in particular conditions of low bacterial counts during refrigerated storage (Santos et al., 2003).

For UHT and sterilized milk processed with a low risk of post-heat treatment contamination, the unwanted presence and even outgrowth of micro-organisms is a rare event and restricted to a few particular endospore formers of which *Bacillus sporothermodurans* is the main cause of concern (Scheldeman et al., 2006). However, the most important spoilage problem of UHT and sterilized milk and related UHT dairy products (cream, custard, evaporated condensed milk, chocolate milk, flavored milk, infant formula, drinks based on milk) is caused by enzymes which resist UHT treatment and which are mainly of bacterial origin. These bacteria are psychrotolerants such as pseudomonads which are able to grow and to produce these thermotolerant enzymes in the cooled raw milk before heat processing. Also milk powder, which is made with a low, medium or high heat process, can contain these thermotolerant enzymes and as a consequence products made with these contaminated milk powders (e.g., desserts, ice mixes, chocolate, confectionery, reconstituted milk) can show a similar spoilage mechanism.

With the current world production and distribution systems of the food industry, there is a real need for high-quality products with ESL. The dairy industry must constantly optimize and improve the processes that result in products that meet business and consumers’ demands and which can be exported over long distances and sometimes in unfavorable storage conditions without loss of quality. Despite the further development of the dairy industry in the last century, premature spoilage of milk continues to be a problem and causes considerable environmental and economic losses (Vanetti, 2009). These economic losses are caused by the direct costs of recalls of products and indirectly by the image damage to the companies concerned. A recall of consumer milk typically occurs upon complaints of gelation or sedimentation of milk or sensory deviations before the shelf life has expired. Such a recall depends on the size of the batch of processed raw milk. Recall costs involve the direct sales costs of the recalled goods but also administrative and logistical costs. It can be estimated that total costs are a multitude of the direct costs related to a recall. If the recall pertains to a product containing milk powder, the recall costs may be greater than for consumer milk.

A safe, abundant, and high-quality milk supply should be the goal of every dairy producer in the world. To achieve this, the control strategies must start at the farm and continue throughout processing. To meet increased raw milk quality standards, producers must adopt practices that reduce mastitis and bacterial contamination of raw milk. Raw bovine milk and dairy products are characterized by a wide microbial biodiversity, with more than 150 species identified (Delbès et al., 2007; Vithanage et al., 2014). Various microbial consortia of raw milk have been studied, particularly in relation to the geographical origin in order to maintain and exploit the microbial diversity in traditional dairy products (Boubendir et al., 2016). Furthermore, von Neubeck et al. (2015) estimated that about 18% of isolates from raw milk belong to hitherto unknown species, indicating that a large fraction of the milk microbiota is still unexplored. Nowadays, studies of the structure and the dynamics of milk microbiota based on a polyphasic taxonomic approach as well as culture-independent methods have advanced knowledge. In this review, the most recent findings on the biodiversity of the milk microbiota contributing to spoilage of milk and dairy products with a long shelf life at mostly ambient temperature will be discussed. The biodiversity will be dealt with on the taxonomic and enzymatic level, along with the specific technological problems caused by the heat-resistant or thermotolerant enzymes (peptidases, lipases, and phospholipases) and possible control strategies.

## Sources of Contamination of Raw Milk

Milk is supposed to be sterile in healthy udder cells. When it leaves the udder it normally contains low numbers of microorganisms, typically ranging from several hundred to a few thousand colony-forming units per milliliter (CFU/mL). De Jonghe et al. (2011) measured a total aerobic plate count around 10,000 cfu/ml at the beginning of storage of the raw milk. But in some countries, raw milk may occasionally be contaminated with much higher numbers of up to 10^7^ CFU/mL at the beginning of storage (Machado et al., 2015) depending on the hygienic conditions under which the milk is obtained.

The diversity of raw milk contamination is influenced by handling factors at the production farms. Numerous microorganisms, including bacteria, yeasts, and molds constitute the complex ecosystem present in milk and dairy products. At the farm level, microbial contamination of bulk tank milk occurs via three main sources: bacterial contamination from the external surface of the udder and teats, from mastitis organisms from within the udder and from the surface of the milking equipment (Murphy and Boor, 2000). Air, water, feed, grass, feces, and soil could also represent important sources of milk contamination. Vacheyrou et al. (2011) proved that most of the fungi and bacteria found in milk were also present in the barn and milking parlor environments.

The teat surface may be an important route of milk contamination (Vacheyrou et al., 2011) and a positive association has been found between udder hygiene score and bacterial counts in bulk tank milk (Elmoslemany et al., 2010). Verdier-Metz et al. (2012) have noted that the composition of the microbiota on teat skin varied qualitatively and quantitatively from one farm to another. This can be attributed to different factors including the farming practices as well as dairy breed, type of feed, type of barn, milking system and quality of milking hygiene practices (Monsallier et al., 2012). Mallet et al. (2012) have shown that teat care has more influence on the composition of technologically relevant microbial groups than on the composition of other groups such as *Pseudomonas* and other Gram-negative bacteria in milk.

Braem et al. (2012) showed that the contaminant microbiota of udder is influenced by the infection status of the udder quarters. The contaminant microbiota from non-infected quarters consists predominantly of *Aerococcus*, *Acinetobacter*, *Corynebacterium*, *Jeotgalicoccus*, *Kocuria*, *Staphylococcus*, and *Bifidobacterium* genera (Ryser, 1999; Jost, 2007; Braem et al., 2012). Besides the diversity of bacterial genera found on the teat apex of dairy cows, Braem et al. (2012) highlighted the presence of a variety of different species of *Corynebacterium* and *Staphylococcus*. The udder of dairy cows may be a source of commensal skin associated bacteria, opportunistic pathogenic bacteria, and mastitis-causing pathogens, which could be found in raw milk.

There are some conflicting results on the importance of udder hygiene in the contamination of milk, depending on the type of microorganism. Masiello et al. (2014) showed that the percentage of dirty udders in the milking parlor combined with the herd size is significantly associated to the raw milk quality (related to psychrotrophic spore formers) and the shelf life of pasteurized milk. On the other hand, Richard et al. (1981) observed that intensive washing of milking equipment and udder preparation (individual washings) results in raw milk that contains a majority of spoilage microorganisms, such as coliforms and *Pseudomonas* spp. In contrast, minimal hygiene around the udder yields raw milk with a majority of useful cheese-making microorganisms including salt-tolerant microbiota such as *Micrococcus*, *Arthrobacter*, *Microbacterium*, *Brevibacterium*, and *Staphylococcus* spp. (Lafarge et al., 2004) and the lactic acid bacteria (LAB) (Desmasures et al., 1997b).

Regarding milking hygiene practices, the cleanliness of milking equipment and storage tanks could affect the introduction and increase in the number of pathogens and other milk quality-affecting bacteria. The contaminant microbiota may persist in water, teat cups, and milking equipment over time indicating a continuous source of microorganisms (Flach et al., 2014; Nucera et al., 2016). This persistence can possibly be explained by biofilm formation and consequent high resistance to disinfection. The milking machine type influences the level of microorganisms in milk, suggesting that these machines are microbiological reservoirs (Mallet et al., 2012). It is well established that the milking machine and storage equipment are commonly colonized by bacterial biofilms (Boari et al., 2009; Marchand et al., 2012; Teh et al., 2012, 2014a). In fact, strains belonging to *Pseudomonas fluorescens*, *Staphylococcus aureus*, *Bacillus licheniformis*, *Serratia liquefaciens*, *Hafnia alvei*, and *Streptococcus uberis* isolated from raw milk tankers are capable of producing biofilms on stainless steel (Teh et al., 2011). In addition to the specific ability of each species or strain, the bacterial adhesion may be affected by the surface roughness and the effectiveness of cleaning processes (Cais-Sokolinska and Pikul, 2008; Vilar et al., 2012). Although biofilm formation within a tanker is of concern, the risk of biofilm development seems to be greater in other areas of a dairy plant (Darchuk et al., 2015).

The quality of water used for cleaning process could affect the contamination level on the surfaces and equipment. A farm water purification system is advised (Garcia Barbero, 1998). In a study performed by Vilar et al. (2008), the bulk-tank bacterial count increased by 12% when non-chlorinated water was used for cleaning. Drinking water and cow feed (including grass silage, soy bean meal, and pasture) are other possible routes for raw milk contamination with *Pseudomonas* spp. through fecal excretion and subsequent contamination of the udder (Marchand et al., 2009a).

Microbial contamination could be transferred from the barn environment, including settled dust and hay, to raw milk. Despite the massive microbiota in the barn, less than a third of this bacterial diversity may be found in milk samples, indicating that there is a partial barrier between barn and milk (Vacheyrou et al., 2011). Differences in housing strategy and feed formulation may contribute to the composition of the bacterial population of milk. Coorevits et al. (2010) demonstrated a greater number of thermotolerant spore-forming bacteria in milk from conventional dairy farms than from organic dairy farms. In the latter, a higher occurrence of *Bacillus cereus* was attributed to differences in housing strategy. It remains to be investigated whether operational management could also influence other spoilage bacteria.

The wide variety of sources of contamination contribute to the complexity of raw milk microbiota; further investigation is clearly needed to fully understand the routes of raw milk contamination with particular spoilage bacteria like pseudomonads and subsequent control of these microbial sources.

## Composition of Raw Milk Microbiota and the Impact of Cold Storage

To understand how the specific spoilage microbiota evolves in raw milk, it is important to know the dynamics of its total microbial composition as a function of the cooled storage time. As raw milk is contaminated during the milking process, several studies have been performed with the aim of identifying the predominant microbiota present in raw cow’s milk immediately after milking (**Table 1**). Although the region where milk samples were collected and the methods used for isolation and identification could influence the results obtained for the predominant microbiota in fresh raw milk, the genus *Lactobacillus* was identified within the dominant microbiota in French raw milk using agar-based methods (Vacheyrou et al., 2011) and 16S rRNA gene-based analyses (Delbès et al., 2007). *Lactobacillus delbrueckii* spp. *lactis* and *Lactobacillus casei* as well as *Lactococcus lactis*, also found in Italian milk samples, have a particular importance within the dairy industry (Bertazzoni Minelli et al., 2004; Quigley et al., 2013). *Propionibacterium freudenreichii* and *Corynebacterium* have been detected in raw milk in a recent study (Quigley et al., 2013). Therefore, the technologically relevant Gram-positive bacteria represent the most prevalent bacterial populations in fresh raw milk obtained from healthy cows and under hygienic conditions. However, according to studies described in **Table 1**, some species from the genera *Staphylococcus* and *Streptococcus* are often detected in fresh raw milk as well as members of the Clostridiales. While *Staphylococcus* and *Streptococcus* have been associated with mastitis infections (Todhunter et al., 1995; Zadoks et al., 2001), *Clostridium lituseburense* and *Clostridium glycolicum*, predominant in cow manure and dairy wastewater, are associated with environmental contamination (Liu et al., 2009; St-Pierre and Wright, 2013, 2014).

**Table 1:** Predominant bacterial groups found in fresh raw milk from different countries using culture-dependent and culture-independent methods.

Country	Predominant groups		Reference
	Culture-dependent methods	Culture-independent methods	
France	Halophilic		Michel et al., 2001
	Mesophilic Aerobic		
	*Pseudomonas*		
	*Staphylococcus*		Vacheyrou et al., 2011
	*Acinetobacter*		
	*Corynebacterium*		
	*Streptococcus*		
	*Lactobacillus delbrueckii* ssp. *lactis*		
	*Lactobacillus paracasei*		
	*Lactobacillus plantarum*		
	*Propionibacterium freudenreichii*		
		*Clostridium* spp.	Delbès et al., 2007
		*Clostridium lituseburense*	
		*Clostridium glycolicum*	
		*Lactococcus lactis*	
		*Lactobacillus casei*	
		*Streptococcus dysgalactiae*	
		*Turicibacter sanguinis*	
		*Ralstonia picketti*	
		*Arthrobacter arilaitensis*	
		*Corynebacterium confusum*	
Italy		*Staphylococcus aureus*	Giannino et al., 2009
		*Enterococcus* spp.	
		*Enterococcus faecalis*	
		*Leuconostoc lactis*	
		*Macrococcus caseolyticus*	
		*Lactococcus lactis*	
		*Rothia* spp.	
The United States		*Staphylococcus*	Kable et al., 2016
		*Streptococcus*	
		*Corynebacterium*	
		Clostridiales	

Cold storage of raw milk is normally applied to reduce the growth of most bacteria. In general, milk is not directly processed after milking and it is stored up to 4 days depending on the legislation of the country (Perin et al., 2012). In dairy processing plants, additional storage until processing is possible (von Neubeck et al., 2015). In an effort to reduce the total aerobic plate count of raw milk, a lower storage temperature (1 to 4°C) is applied, leading to the perception that raw milk could be stored for a longer period before further processing (De Jonghe et al., 2011). Cold storage creates selective conditions for growth of psychrotrophs and considerable changes in the bacterial communities will occur.

The microbiota of fresh raw milk has been described as predominately Gram-positive, but after cold storage, Gram-negative species become predominant in most studies (**Table 2**). The differences in the predominant microbiota after refrigeration can be explained by the variety of cold storage conditions and the original raw milk microbiota in each study. The dominant Gram-negative microbiota found in raw milk stored at cold temperatures belong to the genera *Pseudomonas*, *Stenotrophomonas*, *Aeromonas*, *Hafnia*, *Acinetobacter*, *Serratia*, and *Chryseobacterium* and Gram-positives include *Bacillus*, *Paenibacillus*, *Lactococcus*, *Enterococcus*, *Lactobacillus*, *Staphylococcus*, *Streptococcus*, and *Microbacterium*. Some genera were detected less frequently in raw milk such as *Kocuria* (Lafarge et al., 2004; Mallet et al., 2012; Hanamant and Bansilal, 2013) and *Facklamia* (Rasolofo et al., 2010, 2011).

**Table 2:** Predominant bacterial groups found in raw milk samples in different countries using culture-dependent and culture-independent methods after cold storage.

Country	Predominant groups		Storage conditions	Reference
	Culture-dependent methods	Culture-independent methods		
Algeria	*Stenotrophomonas rhizophila*		4°C for 7 days	Boubendir et al., 2016
	*Stenotrophomonas maltophilia*			
	*Chryseobacterium indologenes*			
	*Lactobacillus pentosus*		4°C for 10 days	
	*Lactobacillus plantarum*			
	*Acinetobacter guillouiae*		4°C for 21 days	
Australia	*Pseudomonas fluorescens*		2°C for 10 days	Vithanage et al., 2016
	*Bacillus cereus*			
	*Bacillus weihenstephanensis*			
	*Bacillus circulans*			
	*Pseudomonas*		4–10°C for 10 days	
	*Acinetobacter*			
	*Hafnia*			
	*Bacillus*			
	*Lactococcus*			
	*Microbacterium*			
Brazil	*P. fluorescens*		4°C for 2 days	Pinto et al., 2015
	*Pseudomonas putida*			
	*Pseudomonas stutzeri*			
	*Serratia liquefaciens*			
	*Serratia odorifera*			
	*Bacillus amyloliquefaciens*			
	*Bacillus subtilis*			
	*Bacillus* sp.			
	*Paenibacillus alvei*			
	*Paenibacillus maceran*s			
	*Lactococcus lactis subsp. cremoris*			
	*Lactococcus* sp.			
	*Enterococcus faecalis*			
	*Enterococcus faecium*			
	*P. fluorescens*		NI	Arcuri et al., 2008
	*Acinetobacter* spp.			
	*Aeromonas hydrophila*			
Canada		*Acinetobacter* sp.	4°C for 3 days	Rasolofo et al., 2010
		*Acinetobacter calcoaceticus*		
		*Staphylococcus aureus*		
		*Staphylococcus equorum*		
		*Facklamia tabacinasalis*		
		*Enterococcus faecium*		
		*Lactococcus lactis*		
		*Streptococcus uberis*		
		*Pseudomonas fluorescens*	4°C for 7 days	
France	*Staphylococcus haemolyticus*		2–4°C for 24–48 h	Mallet et al., 2012
	*Staphylococcus aureus*			
	*Staphylococcus saprophyticus*			
	*Staphylococcus hominis*			
	*Staphylococcus epidermidis*			
	*L. lactis*			
	*Enterococcus faecalis*			
	*Kocuria rhizophila*			
	*Stenotrophomonas maltophilia*			
	*Acinetobacter johnsonii*			
	*Pseudomonas*		5°C for 24–48 h	Richard and Houssu, 1983
	*Fravobacter-Cytophaga*			
	*Coliforms*		8–12°C for 24–48 h	
	*Pseudomonas*			
	*Pseudomonas*		4°C for 24 h	Desmasures et al., 1997a
	*Lactococci*			
	*Micrococcaceae*			
Germany	*Pseudomonas proteolytica*		4-5°C for 3–4 days	von Neubeck et al., 2015
	*L. lactis*			
	*Acinetobacter* sp. nov.			
Israel		*Pseudomonas*	4°C for 22–102 h	Raats et al., 2011
		*Acinetobacter*		
		*Staphylococcus*		
The United States	*Pseudomonas*		7°C for 12–18 h	Jayarao and Wang, 1999
	*Klebsiella*			
	*Enterobacter*			
	*Escherichia*			
Tunisia	*Pseudomonas*		4°C for 24–96 h	Mankai et al., 2003
	*Aeromonas*			

*NI – The storage conditions were not informed*.

According to Lafarge et al. (2004), *L. lactis* was the most frequently detected species in French raw milk samples, along with some *Staphylococcus* species. After incubation of the raw milk at 4°C for 24 h, the majority of samples showed decreased representation of *L. lactis* and minority species such as *Lactobacillus plantarum* and *Lactobacillus pentosus* were outcompeted by other species. Despite a wide variance of the predominant groups found in raw milk after cold storage, Lafarge et al. (2004) noted an emergence of psychrotrophic bacteria such as *Listeria* spp. and *Aeromonas hydrophila*. Raats et al. (2011) examined the prevalence of Gram-positive and -negative bacteria in farm-collected raw milk samples and cold-stored dairy plant samples. The farm samples revealed a prevalence of Gram-positive bacteria (bacilli, clostridia, and actinobacteria) while the dairy plant samples were characterized primarily by Gram-negative species (Gammaproteobacteria). In a 16S rDNA based sequencing approach and despite variance in the predominant microbiota according to time and temperature of raw milk storage, Kable et al. (2016) demonstrated that raw milk microbial communities in tanker trucks in California (USA) were similar to each other even when samples were collected from different farms, transported to different locations and sampled at different times of the year. Surprisingly, these authors showed that the core microbiota (i.e., taxa present in all milk samples) of raw milk, consisting of 29 taxonomic groups, contained high proportions of *Streptococcus* and *Staphylococcus* and unidentified members of Clostridiales, but not *Pseudomonas*, which was present in relatively high proportions in some of the milk tested but entirely absent from two of the tankers examined. They also observed that *Pseudomonas*, along with psychrotrophic species of the genera *Lactococcus*, *Streptococcus* and *Acinetobacter*, tended to be present in relatively higher proportions in dairy plant silos than in the tanker trucks.

The psychrotrophic count, approximately 10% of the total count of mesophilic aerobes immediately after milking performed under hygienic conditions, may reach an average of 90% after cold storage (Sørhaug and Stepaniak, 1997; Catanio et al., 2012). Rasolofo et al. (2010) noted that the biodiversity of raw milk microbiota decreased over the time of cold incubation until psychrotrophic microbiota dominate. However, this group of cold-loving bacteria can represent more than 75% of the initial microbiota of raw milk when collected under conditions of poor hygiene (Hantis-Zacharov and Halpern, 2007; Malacarne et al., 2013).

## The Milk Spoilage Microbiota Producing Heat-Stable Enzymes

While pasteurization inactivates most but not all of the bacteria found in raw milk, UHT treatment renders a product free of microorganisms in the vegetative state. However, several of the psychrotrophic microorganisms may secrete hydrolytic enzymes, which can be heat resistant from pasteurization up to UHT level. On the one hand, these hydrolytic enzymes may be an important tool for the food (dairy) industry as these enzymes may contribute to the development of cheese flavor and texture during ripening (Hasan et al., 2006; Tavano, 2013). On the other hand, the hydrolytic enzymes produced by psychrotrophic bacteria are also widely related to technological problems in milk and dairy products. *Pseudomonas* (mainly the *P. fluorescens* group), *Bacillus*, *Serratia*, and *Hafnia* have strong proteolytic potential while other species of *Pseudomonas* (mainly non-fluorescent pseudomonads), *Bacillus*, *Enterobacter*, and *Acinetobacter* are strongly lipolytic (Hantis-Zacharov and Halpern, 2007). According to the studies listed in **Table 3**, *Pseudomonas* is the predominant spoilage genus isolated from cold raw milk that secretes a heat-stable hydrolytic enzyme. This predominance has been detected at most sampling locations regardless of the approaches used for isolation and identification or time of milk storage.

**Table 3:** Predominant spoilage species isolated from cold raw milk using culture-dependent and culture-independent methods.

Country	Predominant groups		Reference
	Culture-dependent methods	Culture-independent methods	
Australia		*P. fluorescens*	Lo et al., 2016
		*Serratia*	
Belgium	*Pseudomonas lundensis*		Marchand et al., 2009a
	*Pseudomonas fragi*		
Brazil	*Pseudomonas* spp.		Machado et al., 2015
	*Serratia liquefaciens*		
Italy	*Pseudomonas* spp.		Decimo et al., 2014
	*Enterobacter cloacae*		
	*Hafnia alvei*		
	*Serratia marcescens*		
	*Citrobacter freundii*		
Germany		*P. proteolytica*	von Neubeck et al., 2015
		*Pseudomonas* sp. nov. (1)	
		*P. lundensis*	
		*P. fragi*	
		*Acinetobacter*	
Sweden and Norway	*P. fluorescens* biovar I		Ternström et al., 1993
	*P. fluorescens* biovar III		
	*P. lundensis*		
	*P. fragi*		
The United States	*P. fluorescens*		Dogan and Boor, 2003
	*P. putida*		

Studies from the literature agree that *Pseudomonas* is the main genus related to milk spoilage, but within the genus, a diversity of the dominant hydrolytic *Pseudomonas* species isolated from milk samples is observed. Previous older studies focused on *P. fluorescens*, considered the main milk-spoilage species (Makhzoum et al., 1995; Liao and McCallus, 1998; Matselis and Roussis, 1998; Ahn et al., 1999; Kumeta et al., 1999; Woods et al., 2001). However, the taxonomy of the genus *Pseudomonas* is very complex and many new species have been described in the *P. fluorescens* group for which phenotypic methods lack discriminatory power, so the role of *P. fluorescens* in milk spoilage has been overestimated (Marchand et al., 2009a). Even with the application of the sequencing of 16S rDNA and housekeeping genes (*rpoB, gyrB*) and comparison with an up to date in house database for *Pseudomonas*, a recent study on different food matrices could not identify all isolates to the exact species status with many of them classified as closely related to a known species (referred to as the species name + ‘-like’) (Caldera et al., 2016). In that study, besides the species *P. fragi*(-like) and *P. gessardii*-like known as milk spoilers (Marchand et al., 2009b; De Jonghe et al., 2011), several other species as *P. proteolytica, P. brenneri*, and *P. rhodesiae* were found in raw milk, and *P. peli*-like in pasteurized milk. Mostly after applying culture-independent methods for identifying the spoilage microbiota, other species belonging to *Pseudomonas* genus have been identified and characterized (von Neubeck et al., 2016). The peptidase producer *Pseudomonas lundensis* was isolated from raw milk samples from Belgium (Marchand et al., 2009a,b), from Germany (von Neubeck et al., 2015) and from Brazil (Machado et al., 2015). Two novel species, *Pseudomonas helleri* and *Pseudomonas weihenstephanensis*, isolated from cow milk, were characterized based on genetic, phylogenetic, chemotaxonomic, physiological, and biochemical data (von Neubeck et al., 2016). Other studies have demonstrated the (UHT) heat resistance of enzymes produced by *P. weihenstephanensis*, *Pseudomonas proteolytica*, and *Pseudomonas panacis* (Baur et al., 2015b; Stoeckel et al., 2016a).

*Acinetobacter* (like *Pseudomonas* also member of Gammaproteobacteria) is frequently detected in cold raw milk samples (**Table 2**). Strains belonging to this psychrotrophic genus may produce enzymes (Snellman et al., 2002; Salwan and Kasana, 2013) which could potentially lead to milk spoilage. Although some studies have detected hydrolytic strains of *Acinetobacter* in raw milk samples (Nörnberg et al., 2010; von Neubeck et al., 2015; Vithanage et al., 2016), the heat resistance of these enzymes and the spoilage potential from this genus is not well characterized and requires further investigation. *Chryseobacterium* (previously classified in *Flavobacterium*) also appears as a dominant member of Algerian cold raw milk (**Table 2**) and some species like *Chryseobacterium joostei* have been described recently as showing an even greater spoilage capacity than *P. fluorescens* in milk on the basis of growth rate, proteolytic and lipolytic activity (Bekker et al., 2015, 2016). However, besides proteolytic enzymes being resistant to pasteurization, resistance of these enzymes to UHT is not known.

The wide biodiversity of the microbiota of cold raw milk has led to less frequent reporting of several spoilage species. Although the predominance of *Pseudomonas* is well known, the importance of *Serratia* has been described more recently. Along with strains belonging to *Pseudomonas*, *Serratia* was also detected and characterized as a predominant milk spoiler in Australian, Brazilian, and Italian samples (**Table 3**). Teh et al. (2011) and Cleto et al. (2012) have detected *Serratia* in milk-processing plants and raw milk road tankers, respectively, while Lo et al. (2016) reported that *P. fluorescens* and *Serratia* were responsible for spoilage of raw milk stored at 4°C for 7 days. Lo et al. (2016) also showed that *Serratia* was slightly more dominant than *P. fluorescens* (50% vs. 42%) in a raw milk sample collected in a small Australian farm during the autumn. Besides *Serratia*, other psychrotrophic bacteria belonging to Enterobacteriaceae have been isolated from cold raw milk and have been identified as potential milk spoilers due to heat-resistant enzymes. Tondo et al. (2004) described the extensive coagulation of milk proteins after incubation with the heat-resistant peptidase of *Klebsiella oxytoca*. Other examples of the enteric group often detected in raw milk samples are *H. alvei*, *Hafnia paralvei*, and *Enterobacter aerogenes*, which are not only predominant species in raw milk, but also the most enzymatically active genera (Chen et al., 2011; Vithanage et al., 2016).

Although spoilage microbiota in raw milk is mostly Gram-negative and psychrotrophic, some Gram-positive genera have been highlighted, including *Bacillus, Paenibacillus* (both containing psychrotrophic members) as well as thermophilic *Geobacillus. Paenibacillus polymyxa*, *B. cereus*, *B. licheniformis*, and *Bacillus subtilis* are frequently linked to milk spoilage (Ternström et al., 1993; De Jonghe et al., 2010; Hanamant and Bansilal, 2012; Ranieri et al., 2012; Gopal et al., 2015) together with *Geobacillus thermoleovorans* and *Geobacillus stearothermophilus* (Sadiq et al., 2016). According to De Jonghe et al. (2010) and Sadiq et al. (2016), they are the producers of spoilage enzymes more particularly heat-stable lipase (Vithanage et al., 2016) that may adversely affect the quality of milk powder and dairy products made with milk powder (Chen et al., 2003). At refrigeration temperatures (e.g., 5–7°C), spores without heat activation do not germinate and remain stable in milk (De Jonghe et al., 2010) and unless vegetative cells are present in biofilms on the milking or dairy equipment with the release of spoilage enzymes prior to heat treatment (Chen et al., 2004), it is questionable whether these spore formers play a role in spoilage of milk with long shelf life.

## Heat-Stable Spoilage Enzymes Produced by Psychrotrophic Microorganisms

In general, the majority of psychrotrophic microorganisms isolated from milk have the ability to produce hydrolytic enzymes that break down the major milk constituents such as protein, fat and lecithin (Sørhaug and Stepaniak, 1997; Baur et al., 2015a; von Neubeck et al., 2015). Several peptidases, lipases and phospholipases produced by psychrotrophic bacteria isolated from milk have been described in the literature (Sørhaug and Stepaniak, 1997; Chen et al., 2003; Samaržija et al., 2012). Many of these hydrolytic enzymes are heat resistant and consequently retain part of their activity after conventional heat treatment applied in dairy industries such as pasteurization and UHT treatment. Regarding quality and economic aspects, the thermostable hydrolytic enzymes have the most significant effect in dairies since these enzymes lead to flavor defects and technological problems such as sedimentation and gelation in UHT milk, rancidity and flavor defects in milk powder and cheese during their shelf life (Sørhaug and Stepaniak, 1997).

### The Main Proteolytic Enzymes Found in Raw Milk

The term proteolytic enzyme includes all the hydrolases that act on proteins, or further degrade the fragments of them. A few synonyms of proteolytic enzymes such as peptide-bond hydrolase, peptidase or protease could be found in the literature albeit the International Union of Biochemistry and Molecular Biology (IUBMB) recommend the term peptidase (Barrett, 2001). The main problem of the peptidases secreted by psychrotrophic bacteria is that they are heat-stable, which means that they resist at least pasteurization but it is not always described to what extent these enzymes also resist the higher temperatures of UHT.

AprX is the most studied heat-stable peptidase produced by the microbiota found in raw milk, although other species isolated from milk samples may also produce peptidases different from AprX, such as *Klebsiella oxytoca* (Tondo et al., 2004) or *Serratia liquefaciens* (Decimo et al., 2014). *Bacillus* spp. show more diverse proteolytic activity than *Pseudomonas* spp., and many species may secrete more than one type of extracellular and intracellular peptidase (Nabrdalik et al., 2010). The majority of heat-stable spoilage peptidases found in milk samples maintained at refrigeration conditions are produced by Gram-negative bacteria. This section therefore focuses on heat-stable peptidases from *Pseudomonas* and *Serratia*.

#### Peptidase from *Pseudomonas* Isolated from Milk and Dairy Products

The misidentification within the *P. fluorescens* group and an overestimation of the relevance of *P. fluorescens* in milk and dairy products spoilage has led to a large number of works focused on purification and characterization of heat-resistant peptidase produced by the so-called species *P. fluorescens* (Azcona et al., 1989; Kohlmann et al., 1991; Kim et al., 1997; Liao and McCallus, 1998; Matselis and Roussis, 1998; Costa et al., 2002; McCarthy et al., 2004; Maunsell et al., 2006; Dufour et al., 2008; Mu et al., 2009; Martins et al., 2015; Zhang et al., 2015). Although acknowledging this problem, the species name as described in the, respectively, cited literature will be retained in this review.

The number of the different peptidases produced by the genus *Pseudomonas* is heterogeneous and varies according to the species and the strains (Nicodème et al., 2005). Most of the studies mentioned in the paragraph above show that the strains of *Pseudomonas* spp. isolated from raw milk secrete at least one monomeric peptidase with molecular weight varying between 23 and 56 kDa. The metallopeptidase AprX is the main heat-resistant peptidase in the genus *Pseudomonas* related to milk spoilage targeted in the literature (Liao and McCallus, 1998; Dufour et al., 2008; Marchand et al., 2009b; Baglinière et al., 2013; Martins et al., 2015). This enzyme is mainly secreted by the species *P. fluorescens*, but this peptidase has also been detected in various other species found in raw milk belonging to the genus *Pseudomonas* such as *P. fragi*, *P. tolaasii, P. rhodesiae, P. gessardii, P. proteolytica, P. brenneri*, or *P. chlororaphis* (Martins et al., 2005; Marchand et al., 2009b; Caldera et al., 2016). Although no *aprX* sequence has yet been obtained, *P. lundensis* produces a similar enzyme, as evidenced by a few peptides of *P. lundensis* retrieved by mass spectrometry, which display similarity with the other *Pseudomonas* AprX proteases (Marchand et al., 2009b).

AprX is a peptidase of 45 to 50 kDa encoded by the *aprX* gene located on the *aprX-lipA* operon, which contains eight genes and spans 14 kb (McCarthy et al., 2004). In general, AprX is rich in alanine and glycine residues and poor in cysteine and methionine residues (Dufour et al., 2008). The lack of cysteine residues allows avoidance of steric constraints due to disulphide bonds and increases its flexibility (Matéos et al., 2015). The presence of Ca^2+^ (GGXGXDXUX) and Zn^2+^ (HEIGHTLGLAHP) binding motifs confirms its dependence of divalent-cations (Dufour et al., 2008). The AprX protein is highly conserved within *Pseudomonas* species (76–99% similarity for AprX of *P. fluorescens* group), but is more heterogeneous between species (57–69% similarity for AprX between strains of *P. fluorescens* and *P. fragi*) (Marchand et al., 2009b; Matéos et al., 2015). In addition to the four AprX sequence groups (with one group split into two subgroups) identified within *Pseudomonas* raw milk isolates by Marchand et al. (2009b), a fifth group was added recently including Mozzarella isolates (Caldera et al., 2016).

AprX exhibits activity in a large range of pH (4.5–10) with an optimum activity between 7.5 and 9, which proves that AprX is an alkaline peptidase. AprX generally exhibits activity in a large range of temperatures (0–55°C) with optimal activity between 37 and 47°C (Dufour et al., 2008; Martins et al., 2015; Matéos et al., 2015). Inhibition studies revealed that AprX was inhibited by typical divalent-ion chelators such as EDTA (Ca^2+^ and Zn^2+^ chelator), EGTA (Ca^2+^ chelator), *o*-phenanthroline (Zn^2+^ chelator) while serine peptidase inhibitors (PMSF and leupeptin) did not affect activity of the enzyme (Liao and McCallus, 1998; Dufour et al., 2008; Matéos et al., 2015). It was shown for an alkaline metallopeptidase isolated from a *Pseudomonas* sp. isolated from refrigerated milk, that Ca^2+^ stabilizes the enzyme and improves its activity (Ertan et al., 2015), while Zn^2+^is essential in the active site (Wu and Chen, 2011).

**FIGURE 1:**
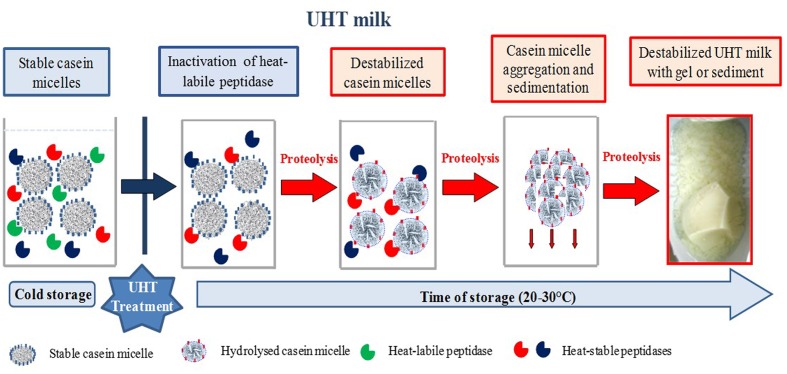
**Hypothetic mechanism of UHT milk destabilization due to casein micelle proteolysis by heat-resistant peptidase during storage at ambient temperature**. The different species and strains of proteolytic psychrotrophic bacteria may produce heat-stable peptidases, which hydrolyze different types of casein. Some heat-resistant peptidases have preferential cleavage sites in hydrophobic areas of casein (red areas) while others hydrolyze preferentially the κ-casein which makes the connection between the hydrophobic core and the hydrophilic medium (blue areas).

AprX may hydrolyze the four types of casein (α_s1_, α_s2_, β, and κ) with a large activity spectrum (Baglinière et al., 2013). Matéos et al. (2015) have shown that cleavage sites are mainly found in hydrophobic areas of casein. The extracellular peptidase produced by *P. fluorescens* hydrolyzes milk caseins preferentially in the following order κ- > β- > α_S1_-caseins (Fairbairn and Law, 1986; Mu et al., 2009; Pinto et al., 2014; Zhang et al., 2015). However, Baglinière et al. (2012) described the preferential proteolysis of β-casein by AprX. This difference in preferential proteolysis between the different studies could be attributed to the differences in the species and strain used. In **Figure 1**, a hypothetical mechanism of UHT milk destabilization due to casein micelle proteolysis by heat-resistant protease during storage at ambient temperature is shown. The intensity of proteolytic activity is dependent on species and strains. Marchand et al. (2009a) and Baglinière et al. (2012) revealed a large heterogeneity, respectively, in the proteolytic activity within the *Pseudomonas* genus and in effect on destabilization and flavor defects of UHT milk inoculated with *P. fluorescens* strains and other *Pseudomonas* species. Caldera et al. (2016) observed a high total proteolytic activity (without prior heat treatment) for all *P. proteolytica* isolates (4 and 12 μmol glycine equivalent/mL, (as measured with the 2,4,6-trinitrobenzenesulfonic acid [TNBS] method), the major part of *P. gessardii*-like isolates (2 and 16 μmol glycine equivalent/mL), and for 36% of *P. fragi*(-like) isolates (5 and 14 μmol glycine equivalent/mL). The high variability of *Pseudomonas* strains regarding the proteolytic activity may be a consequence of heterogeneous enzyme expression, regulation by quorum sensing (QS), effect of temperature, iron content, and bacterial growth phase (Woods et al., 2001; Nicodème et al., 2005; Marchand et al., 2009a).

Although AprX has been reported as the main heat-stable peptidase encountered in *Pseudomonas* spp. isolated from raw milk in several recent studies (Marchand et al., 2009b; Baglinière et al., 2013; Matéos et al., 2015), some authors showed that *P. panacis* and also *P. fluorescens* can secrete another heat-stable peptidase AprA (Maunsell et al., 2006; Baur et al., 2015b). According to Baur et al. (2015b), the peptidase AprA secreted by a strain of *P. panacis* isolated from raw milk was able to withstand a UHT process. In the same study, the authors showed that the peptide sequence of AprA was 98% similar to the peptidase AprX secreted by a strain of *P. fluorescens*. As AprX, AprA is a metallopeptidase of about 50 kDa belonging to the serralysin family and presents in its primary structure the binding motifs for Ca^2+^ and Zn^2+^ (Takahashi, 2013; Baur et al., 2015b). According to Ma et al. (2003), there is a nomenclatural problem in the Apr protease system of *Pseudomonas.* According to those authors, AprA should be considered the main alkaline peptidase and AprX, lacking both the conserved Zn^2+^binding sequence and the glycine-rich motif of AprA, is produced together with AprA by *P. aeruginosa*. However, the alkaline metalloprotease of *P. fluorescens* responsible for milk spoilage was first described as AprX by Dufour et al. (2008) and has been named as such in most studies since then. AprA and AprX produced by the *Pseudomonas* strains responsible for milk spoilage are the same enzyme because of their high sequence similarity and presence of the conserved motifs, while AprX produced by *P. aeruginosa* is a different enzyme.

A recent study conducted by Stuknytë et al. (2016) detected two other thermostable proteolytic bands with molecular masses of approximately 15 and 25 kDa after zymography analysis from *P. fluorescens* PS19 supernatant. The 25-kDa fragment did not show homology to AprX, indicating that this strain is able to secret a heat-stable peptidase other than AprX or AprA.

#### Heat-Stable Peptidase from *Serratia* Isolated from Milk and Dairy Products

The importance of *Serratia* as a milk-spoilage microorganism has been shown recently (Cleto et al., 2012; Decimo et al., 2014; Machado et al., 2015), although previous studies have described and/or characterized peptidases from *S. proteamaculans* (Christensen et al., 2003; Demidyuk et al., 2006; Eom et al., 2014), *S. marcescens* (Matsumoto et al., 1984; Letoffe et al., 1991; Jayaratne, 1996; Romero et al., 2001; Tao et al., 2007; Nam et al., 2013) and *Serratia* sp. E-15 (Hamada et al., 1996).

The number of peptidases produced by *Serratia* is variable. This characteristic could either be species dependent or variable, depending on the method used for peptidase detection. According to Matsumoto et al. (1984), *S. marcescens* kums3958 produced four peptidases as detected by polyacrylamide gel electrophoresis. These peptidases presented a molecular weight of 56, 60, and 73 kDa wherein the 73 kDa-peptidase has been separated in two peptidases after isoelectric focusing. Nevertheless, Romero et al. (2001) detected only two peptidases when *S. marcescens* was inoculated into reconstituted whey. The molecular masses of both peptidases estimated on SDS-PAGE were 53.5 and 66.5 kDa for the metallopeptidase and the serine peptidase, respectively. Those authors did not detect the 73 kDa-peptidase. This result could be explained by the different growth conditions and strains used.

The metallopeptidase from *S. marcescens* S3-R1, which has a molecular weight of approximately 50.3 kDa, has been characterized by Nam et al. (2013). Those authors showed that this peptidase presents its optimal activity at pH 7–9 and at 40–50°C.

Unfortunately, there is no information in the literature about the characterization of the heat-stability of these peptidases. However, Glück et al. (2016) observed, for two strains of *S. marcescens* isolated from raw milk, an extracellular peptidase residual activity of 71 and 91% after a heat-treatment of 95°C for 5 min, highlighting the secretion of heat-stable peptidase by this species. Nevertheless, the authors did not identify the peptidase responsible for this residual activity. Worth noting is that *S. marcescens* is an opportunistic pathogen for human and insects (Ishii et al., 2014; Hagiya et al., 2016), which justifies most studies focused on peptidases produced by this species, while the characterization of *S. liquefaciens* peptidases have been discussed by few authors only (Kaibara et al., 2010, 2012; Machado et al., 2016).

*Serratia liquefaciens* FK01 produces two serralysin-like metallopeptidases (Kaibara et al., 2010, 2012). These peptidases are encoded by *ser1* and *ser2* genes. Both peptidases showed molecular mass of approximately 50 kDa and presented Zn^2+^ binding motif (HEXXHXUGUXH), Ca^2+^ binding motif (GGXGXDXUX), and ABC exporter motif (DXXX) (Kaibara et al., 2010, 2012; Machado et al., 2016). The difference between both peptidases produced by *S. liquefaciens* seems to be heat resistance. According to Machado et al. (2016), only Ser2 withstood the heat treatment of 95°C for 8:45 min. Those authors highlighted that proteolytic activity of Ser2 was highly variable depending on the incubation conditions and on the *S. liquefaciens* strain inoculated into the milk samples.

#### Technological Problems Resulting from the Residual Activity of Peptidases after Heat Treatment

Heat-resistant peptidases can lead to serious problems for the dairy industry. Since *Pseudomonas* has been widely studied, there are several studies focused on technological problems caused by peptidases from *Pseudomonas* (Celestino et al., 1997; Sørhaug and Stepaniak, 1997; Belloque et al., 2001; Datta and Deeth, 2001, 2003; Chen et al., 2003; Baglinière et al., 2013), however, there are no studies yet regarding the consequences of peptidase from *Serratia* in dairy products.

**FIGURE 2:**
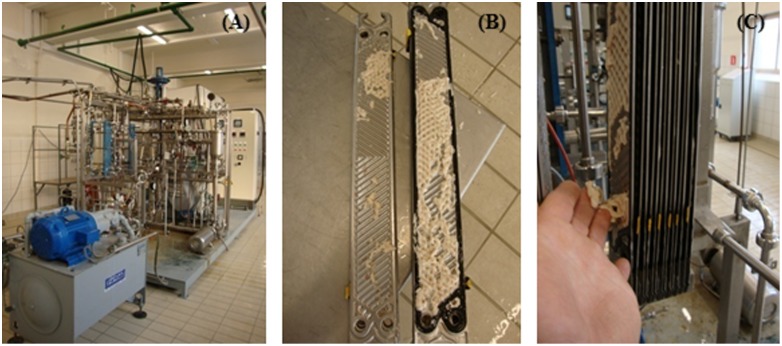
**Clogging of the heating exchanger due to processing of milk spiked with *Pseudomonas* and stored for 5 days at 6.5°C. (A)** UHT Process Pilot Plant (ILVO, Belgium), **(B)** heat exchanger, **(C)** detail of the heat exchanger with clotting of milk.

After raw milk storage for prolonged time, UHT processing can be compromised because of destabilization of the milk, resulting in clogging of the heating exchanger (**Figure 2**). Pinto et al. (2014) showed that α-, β-, and κ-casein from milk inoculated with *P. fluorescens* were completely hydrolyzed after 4 days incubation at 4°C. The proteolysis of casein contributes to destabilization of UHT milk and to protein sedimentation during its storage (Gaucher et al., 2011; Baglinière et al., 2013; Matéos et al., 2015). A visual destabilization of UHT milk by AprX from *P. fluorescens* F was observed after 7 days of storage when 0.2 mg/mL of peptidase had been added in raw milk before UHT treatment (Baglinière et al., 2013). The protein sedimentation could be observed after 2 weeks of storage in UHT milk samples when peptidases from *P. panacis* where inoculated at a final concentration of 1 picokatal/mL (Baur et al., 2015b; one katal of an enzyme is that amount which breaks a mole of peptide bonds per second under specified conditions).

**FIGURE 3:**
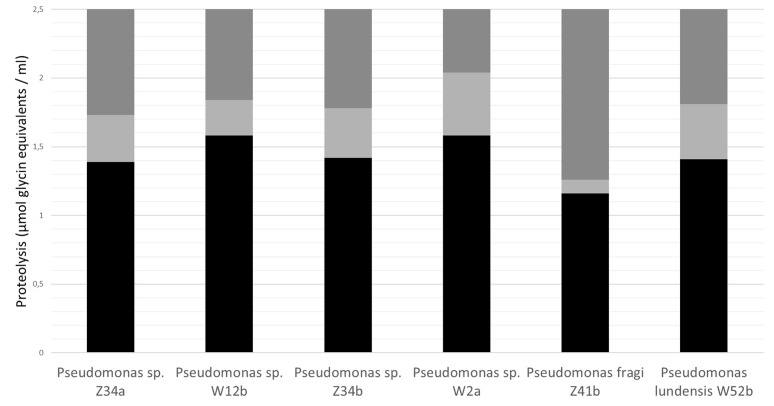
**Correlation between off-flavors and protein hydrolysis by six different *Pseudomonas* peptidase groups in UHT-milk, as described by Marchand et al. (2009b)**. Raw milk was pasteurized, inoculated with one of the *Pseudomonas* strains to a final concentration of 3 log CFU/mL, and stored for 3 to 5 days at 6.5°C until skimming and indirect UHT-processing (5 s at 140°C). Processed milk was aseptically filled in high-density polyethylene bottles of 0.5 L and stored at 37°C to accelerate proteolysis. The six *Pseudomonas* peptidase milk samples were compared with the reference control milk by an experienced taste panel of 35 people at the Institute for Agricultural and Fisheries Research (ILVO), Belgium in a sensorial cabinet equipped with individually partitioned booths. Milk with off-flavor was diluted as follows: (A) *Pseudomonas* milk undiluted, (B) 2/3 *Pseudomonas* milk + 1/3 control milk, (C) 1/3 *Pseudomonas* milk + 2/3 control milk, (D) Control milk undiluted. The taste panel was asked to rank the milk samples (A–B–C–D) according to preference. Statistical evaluation of the results was based on the Rank Test to KRAMER (Kramer, 1960) for α = 0.05. Simultaneously, proteolysis (expressed as μmol glycine equivalents mL^-1^ milk) was determined in each milk dilution (A–B–C–D) by the trinitrobenzenesulfonic acid (TNBS) method (Polychroniadou, 1988). No significant proteolysis off-flavors is indicated by black bars, the uncertainty range by light gray bars (the panel did not reject the milk samples; the lower limit of the bar is determined by the TNBS-value of the most diluted sample that was not rejected by sensory analysis) and the significant proteolysis off-flavors by dark gray bars (panel rejected the milk samples and tasted off-flavors; the lower limit of the bar is determined by the TNBS-value of the least diluted sample). This figure was reproduced from Marchand et al. (2017).

The proteolysis of milk protein can also lead to bitter off-flavor of some dairy products such as UHT milk. This is caused by the generation of hydrophobic peptides by hydrolysis of casein (Chen et al., 2003; Datta and Deeth, 2003). Valero et al. (2001) showed that new flavor and volatile components appeared in skimmed milk samples after 65 days storage related to proteolysis and the Maillard reaction. The proteolytic activity might increase the number of free amino groups, which can participate with the reducing sugars in Maillard reactions (Valero et al., 2001). In UHT-milk spiked with one each of six isolates representing the different *Pseudomonas* peptidase groups, a casein hydrolysis of >1.5 μmol glycine equivalents/mL (as measured with the TNBS method) was the threshold for the taste panel to detect off-flavor, but no clear correlation was found between the onset of off-flavors and the rate of protein hydrolysis (Marchand et al., 2017). The degree of proteolysis (as measured with the TNBS method) of the UHT-milk samples in which off-flavors were significantly tasted, were different for each *Pseudomonas* peptidase under evaluation (**Figure 3**). *P. fragi* peptidase was capable in generating off-flavors after very limited proteolysis (a raise in TNBS-value of 0.15 glycine equivalents/mL). Therefore, it can be speculated that not all *Pseudomonas* peptidases have the same specificity for their casein substrates.

However, a large difference in heat resistant or total proteolytic activity was observed within *P. fragi* by Marchand et al. (2009a) and Caldera et al. (2016), respectively, with only 36% of *P. fragi*(-like) isolates (from diverse sources including dairy and raw milk) showing total proteolytic activity according to the latter authors. Nevertheless, when positive for proteolytic activity, the *P. fragi* group exhibited a significantly higher heat-resistant proteolytic activity than the *P. lundensis* and the *Pseudomonas* spp. group (Marchand et al., 2009a), which suggests that the presence of proteolytic strains of *P. fragi* prior to UHT-processing will severely compromise the shelf life of derived dairy products.

Another technological problem in the dairy industry caused by proteolytic activity from psychrotrophic microorganisms is the yield reduction in cheese manufacturing (Cardoso, 2006; Mankai et al., 2012). Cardoso (2006) showed a reduction of 6.38% in total solids in fresh Minas cheese produced using a raw milk stored for four days under cold temperatures, which promotes psychrotrophic growth.

### Lipolytic Enzymes

Lipolytic enzymes in cow’s milk from endogenous or exogenous sources and the action of these enzymes on the milk substrate is considered as limited by its natural organization in the form of fat droplets (Bourlieu et al., 2012). Undesirable lipolysis of milk and dairy products has not been studied as much as undesirable proteolysis. Exogenous lipases produced by psychrotrophic bacteria can hydrolyze milk fat and release free fatty acids (FFAs), mono- and di-acylglycerols and glycerol. The lipolytic activity of psychrotrophs is species-specific. According to Decimo et al. (2016) bacterial triacylglycerol hydrolysis may occur to a greater or lesser extent, but the type and amount of released FFAs are not easily predictable. The lipolytic activity generates undesirable product flavors such as rancid, unclean, soapy, or bitter, making the product barely acceptable to the consumer (Deeth, 2006).

Among the lipolytic psychrotrophic bacteria, *Pseudomonas* spp. is the predominant Gram-negative group found whereas *Bacillus* spp. is the predominant Gram-positive group (Chen et al., 2003; Decimo et al., 2014; Vithanage et al., 2016). However, other genera isolated from raw milk, may also produce lipolytic enzymes, such as *Serratia*, *Hafnia*, *Acinetobacter, Microbacterium*, and *Enterobacter* (Hantis-Zacharov and Halpern, 2007; Decimo et al., 2014; Vithanage et al., 2016).

Lipolytic enzymes are defined as carboxylesterases that hydrolyse acylglycerols (Jaeger et al., 1994, 1999; Chen et al., 2003). Carboxylesterases can be divided in two groups, the lipase or triacylglycerol acylhydrolases (EC 3.1.1.3) and the esterase or carboxylases (EC 3.1.1.1). Lipases are active at lipid–water interfaces rather than in the aqueous phase and preferentially hydrolyze acylglycerols with more than 10 carbons (>C_10_). Esterases are active in aqueous solutions and are only able to hydrolyze acylglycerols with fewer than 10 carbons (<C_10_) (Anthonsen et al., 1995). Lipase is also capable of hydrolyzing acylglycerols <C10 (Jaeger et al., 1994, 1999) and intact milk fat globules (MFG). Without hydrolysis of the fat globules, the lipolytic enzyme does not have access to triglycerides (Deeth, 2006). In terms of spoilage potential, this difference between lipase and esterase hydrolysis could explain most of the lipolytic enzymes studied are secreted lipase. Another enzyme able to hydrolyze MFG is phospholipase. However, this enzyme cannot hydrolyze triacylglycerol. Therefore, this review focuses on lipase secreted by the genera *Pseudomonas* and *Bacillus*, particularly heat-stable lipase.

#### Lipase from *Pseudomonas* Isolated from Milk and Dairy Products

Among the lipolytic species of *Pseudomonas*, *P. fluorescens* is the species more often found in raw milk. Nevertheless, some recent studies showed the presence of lipolytic strains of *P. aeruginosa*, *P. putida*, *P. fragi*, and *P. gessardii*-like in raw milk or *P. peli*-like in pasteurized milk (Munsch-Alatossava et al., 2013; Decimo et al., 2014; Caldera et al., 2016). *Pseudomonas* spp. produce a large variety of lipolytic enzymes classified into six groups corresponding to family and subfamily (Arpigny and Jaeger, 1999; Chen et al., 2003). However, most of lipases secreted by the species found in raw milk such as *P. aeruginosa*, *P. fluorescens*, and *P. fragi* belong to the sub families I.1 and I.3 (Arpigny and Jaeger, 1999). Most of them have specificity for the sn-1 and sn-3 positions of triacylglycerols, and some hydrolyze diacylglycerols and monoacylglycerols faster than triacylglycerols (Chen et al., 2003). The classification of these enzymes is based on their amino acid homologies and some biological properties. These lipases present the consensus pentapeptide G-X-S-X-G in the amino acid sequence, corresponding to the catalytic site.

Subfamily I.1 corresponds to the secreted lipases with a molecular weight of approximately 30 kDa, which present two aspartic residues involved in the Ca^2+^ binding site (Arpigny and Jaeger, 1999). These lipases are mainly secreted by *P. aeruginosa* and *P. fragi* (Arpigny and Jaeger, 1999). However, the species *P. fluorescens* is also able to secrete a lipase belonging to this subfamily (Beven et al., 2001). Subfamily I.3 corresponds to lipase with a molecular mass of 50 to 65 kDa. The most studied lipase from this group is LipA from *P. fluorescens* encoded by the *lipA* gene located in the same operon as the peptidase AprX, the operon *aprX-lipA.* Similar to the peptidases Ser2 and AprX, this lipase presents in the amino acid sequences the binding motif to fix Ca^2+^ suggesting the need of this ion for its stability. The lipases of *P. fluorescens* 33 (Kumura et al., 1993a,b), *P. fluorescens* 041 (Martins et al., 2015), *P. fluorescens* SIK W1 (Son et al., 2012) isolated from milk and one of the lipases of *P. fluorescens* C9 (Beven et al., 2001) belong to this family.

Numerous older studies have shown the heat-stability of the lipolytic activity of *Pseudomonas* species. Law et al. (1976) showed that after a heat treatment of 63°C during 30 min in raw milk, the extracellular residual lipolytic activities of various strains of *Pseudomonas* isolated from raw milk were 55 to 100%. Fitz-Gerald et al. (1982) observed that lipases from *Pseudomonas* isolated from raw milk presented 75 to 100% of residual lipolytic activity after heating skim milk at 100°C for 30 s. Moreover, Andersson et al. (1979) reported a *D*-value of 23.5 min (calculated time required for a 90% reduction of the initial activity) for *P. fluorescens* SIK W1 lipase after heat-treatment of skim milk at 100°C. As described by Fox and Stepaniak (1983), lipase from *Pseudomonas* seems to be more heat-stable in synthetic milk salt solutions than in phosphate buffer. This better heat-stability in milk salt solutions is probably due to the presence of calcium (Andersson et al., 1979). Recent studies, however, show that not all lipases secreted by the genus *Pseudomonas* are heat-stable. The residual lipase activity of the purified LipM of *P. fluorescens* 041 isolated from Brazilian raw milk was only 25.4% after heat-treatment of 72°C for 20 s in buffer (Martins et al., 2015). It is noteworthy that Vithanage et al. (2016) showed that more than 30% of *Pseudomonas* strains isolated from raw milk presented 50 to 75% of residual lipase activity after a heat treatment of 4 s at 142°C (UHT treatment). Those authors observed more strains producing heat-stable lipases than strains producing heat-labile lipases among *Pseudomonas* strain isolated from raw milk.

These studies confirm that many lipases from the genus *Pseudomonas* can resist heat-treatment used in dairy industries such as pasteurization and/or UHT treatment. No heat treatment is available that may inactivate these lipases without altering the milk’s sensory and nutritional qualities.

#### Lipase from *Bacillus* spp. Isolated from Milk and Dairy Products

The presence of the thermophilic species *Geobacillus stearothermophilus* as a lipolytic enzyme producer in raw milk and milk powder has been reported by various authors (Chopra and Mathur, 1984, 1985; Chen et al., 2004). The principal characteristic of *Bacillus* lipases is the substitution of the first glycine by alanine in the conserved pentapeptide A-X-S-X-G (Arpigny and Jaeger, 1999). Most *Bacillus* lipases show the highest catalytic activities at temperatures ranging from 60 to 75°C (Chen et al., 2003). The lipase of *Bacillus* can be classified in two groups: subfamilies I.4 and I.5. The lipase of *B. subtilis* (molecular mass about 20 kDa) is the smallest true lipase known from bacteria found in raw milk (Arpigny and Jaeger, 1999). This lipase, belonging to subfamily I.4, is also secreted by *B. licheniformis*, frequently encountered in raw milk as a lipolytic enzyme producer (Baur et al., 2015a). With a molecular mass of 45 kDa, the lipase secreted by the species *G. thermocatenulatus* and *G. stearothermophilus* belongs to subfamily I.5 and shows optimal activity at pH 9.0 and 60–65°C (Schmidt-Dannert et al., 1996; Kim et al., 1998; Arpigny and Jaeger, 1999).

As described above for *Pseudomonas*, many *Bacillus* (or *Geobacillus*) lipases remain stable during heat-treatments used in dairy industries (pasteurization and/or UHT treatment) and can therefore affect milk and dairy products during storage. Chen et al. (2003) calculated a *t*_1/2_ of 690 min at 70°C in buffer at pH 7.0 for the lipase produced by a strain of *G. stearothermophilus*. Considering all lipases secreted by *Bacillus*, lipases from strains isolated from milk powder production presented a higher residual activity after pasteurization at 72°C for 2 min in milk (Chen et al., 2004). In addition, a recent study showed that more than 38% of *Bacillus* strains isolated from raw milk presented 50 to 75% of residual lipase activity after a heat treatment at 142°C for 4 s (Vithanage et al., 2016).

#### Phospholipase C

The production of different phospholipases has been reported for Gram-negative and Gram-positive psychrotrophs (Sørhaug and Stepaniak, 1997). The phospholipase most studied is phospholipase C, which can be either hemolytic or non-hemolytic. Phospholipase C activity has been detected in the genera *Pseudomonas*, *Bacillus*, *Serratia*, *Hafnia*, *Acinetobacter*, and *Microbacterium* in raw milk by Vithanage et al. (2016). However, according to De Jonghe et al. (2010), in the genus *Bacillus* only the species *B. cereus* is able to produce the phospholipase C. The presence of this enzyme was not observed for the species *B. licheniformis* or *B. subtilis*. This enzyme is particularly heat stable (Sørhaug and Stepaniak, 1997) and disrupts the integrity of the MFG membrane (Craven and Macauley, 1992; Shah, 1994).

The phospholipase C of *P. fluorescens* is well known as a heat-stable enzyme, presenting high residual activity after pasteurization and UHT treatment (Sørhaug and Stepaniak, 1997). Vithanage et al. (2016) observed that about 25% of *Bacillus* and *Pseudomonas* strains isolated from raw milk presented 50 to 75% of residual phospholipase C activity after heat-treatment of 140°C during 4 s.

#### Technological Problems Resulting from the Residual Activity of Lipases after Heat Treatment

The presence of bacterial lipase could affect the quality of fluid milk, dry whole milk, cheese, and butter (Sørhaug and Stepaniak, 1997; Chen et al., 2003). However, it seems that the modifications induced to milk lipids are highly dependent on the lipase specificity and also on the fat condition. Excessive shaking, addition of air, repeated thermal shocks, and homogenization, all of which can occur at different stages of production and processing, adversely affect the integrity of the fat globule, modify the interfaces between the fat and non-fat phase and lead to an increase of lipolysis (Kim et al., 1983). The action of lipase on milk fat can release short-chain fatty acids (C4:0 to C8:0), medium-chain fatty acids (C10:0 and C12:0) and long-chain fatty acids (C14:0 to C18:0). Short-chain fatty acids (e.g., butyric, caprylic, and caproic acids) have strong flavors and can impart unpleasant flavors variously known as rancid, bitter, butyric, unclean, astringent, or ‘lipase’ (Deeth, 2011), whereas medium-chain fatty acids are responsible for a soapy taste (Chen et al., 2003). Long-chain fatty acids contribute little to flavor. Moreover, as described by Chen et al. (2003), an oxidized flavor can be generated by the oxidation of free unsaturated fatty acids to aldehydes and ketones. Due to presence of heat-stable lipases in raw milk, theses undesirable flavors, such as rancidity, can occur in UHT-milk (Adams and Brawley, 1981). The mono- and di-acylglycerols which are the other products of lipase action have surface-active properties that can affect some products such as steam-foamed milk used in coffee-based drinks (Deeth, 2011).

Whole milk powder can be also affected by residual heat-resistant lipase, because most enzymes are more stable when water activity decreases. Indeed, some authors showed that lipase of *P. fluorescens* in spray-dried powder did not lose activity at 20°C for up to 60 days (Shamsuzzaman et al., 1986). According to Andersson (1980), lipases retain more activity than peptidases in milk powder during prolonged storage. Moreover, residual lipase activities may be detected when dry whey products and skimmed milk powder are added as ingredients to fatty products (Stead, 1986).

### Lipase and Peptidase Regulation

Understanding of the regulation of peptidases and lipases produced by psychrotrophic bacteria in milk samples is still limited. Compared to the other psychrotrophic genera, the regulation of enzymes secreted by the genus *Pseudomonas* has been the most studied, especially the operon *aprX-lipA* regulation. However, the complex production process of these two enzymes is not completely understood. The following section will be focused on the regulation of extracellular enzymes produced by the genus *Pseudomonas*. The factors involved in this regulation are described briefly.

Many factors are involved in the lipase and peptidase production by psychrotrophic bacteria, such as temperature (Burger et al., 2000; Nicodème et al., 2005), phase of growth (Chabeaud et al., 2001; van den Broeck et al., 2005), QS (Givskov et al., 1997; Christensen et al., 2003; Juhas et al., 2005; Liu et al., 2007; Pinto et al., 2007) or iron content (McKellar, 1989; Woods et al., 2001).

In *P. fluorescens*, enzyme production seems to be strongly related to cell density. According to Bai and Rai (2011), the production of extracellular peptidases in *P. fluorescens* is associated with the high cell density that is typically encountered at the end of the exponential phase of growth. One hypothesis is that this regulation by cell density may be mediated by QS. Indeed, bacteria may communicate by QS using signaling molecules called *N*-acyl-homoserine lactones (AHLs). These molecules are produced by numerous Gram-negative bacteria such as *Pseudomonas* (Liu et al., 2007) or *Serratia* (Givskov et al., 1997) and are implicated in the genetic control of a wide range of phenotypic attributes such as cell differentiation, biofilm formation, sporulation, toxins, and enzyme secretion (De Oliveira et al., 2015). Their production is strongly dependent on a specific cell density (Fuqua et al., 1994; Liu et al., 2007). A remarkable seasonal variation on heat resistant proteolytic activity of *Pseudomonas* strains from raw milk was observed (Marchand et al., 2009a). This effect could be related to growth rate: in milk samples with proteolytic bacteria, the proteolytic psychrotolerant counts were significantly higher in samples collected in winter than in summer and winter isolates displayed better growth characteristics and peptidase production than the summer isolates.

Pinto et al. (2007) showed that more than 80% of psychrotrophic proteolytic strains isolated from cooled raw milk were able to produce AHLs in raw milk and pasteurized milk, suggesting that QS may play a role in the spoilage of milk. Liu et al. (2007) reported that the proteolytic activity of the strain 395 of *P. fluorescens* was stimulated by the addition of AHL. Those authors concluded that the *aprX* gene was regulated at a transcriptional level by AHL during the end of the exponential phase of growth. In contrast, Pinto et al. (2010) did not observe any effect on the growth and proteolytic activity after adding synthetic AHL in the culture of the proteolytic strain *P. fluorescens* 07A that secretes AprX but does not produce AHL. The extracellular peptidase activity was detected only when the cell population reached 10^8^ CFU/mL. They concluded that peptidase activity of this strain was not regulated by QS via AHLs but could be related to cell density. The regulation of enzyme production by QS via AHLs seems thus to be strain dependent in the species *P. fluorescens*. According to Siddiqui et al. (2005), in *P. fluorescens* CHA0, the expression of *aprA* gene seems to be also cell density dependent. In addition, the authors showed that the expression of this protease was positively regulated by the two-component system GacS/GacA, which controls the expression of secondary metabolism and protein secretion in a wide variety of bacterial species.

Relating to other genera implicated in milk spoilage, the relationship between QS, cell density and enzyme production has been observed among members of the *Serratia* genus. It seems that the operon *slaA-lipB* of *S. proteamaculans*, which is required for the secretion of several unrelated and potentially food-quality-relevant proteins and the exoenzyme production and its homolog in *S. liquefaciens*, is under the transcriptional control of QS (Givskov et al., 1997; Christensen et al., 2003). Christensen et al. (2003) demonstrated that the activities of several exoenzymes including peptidases from *S. proteamaculans* B5a are affected by *N*-(3-oxo-hexanoyl)-L-homoserine lactone, which is a signal molecule of QS system in Gram-negative bacteria. Although Ser2 is a heat-resistant peptidase that may compromise milk product quality, no information about regulation of Ser2 expression is available.

Numerous studies show that the enzymes of *P. fluorescens* are regulated by the temperature of growth. Optimal peptidase production occurs when the temperature of growth is slightly above the optimal temperature of growth, while above this temperature peptidase production is severely repressed (Burger et al., 2000; Woods et al., 2001; McCarthy et al., 2004). In contrast, optimal lipase production occurs when the temperature of growth is well below the optimal growth temperature, suggesting the contribution of low temperature-dependent regulation system (Andersson, 1980; Merieau et al., 1993; Woods et al., 2001). However, a study carried out by Woods et al. (2001) showed that the temperature does not regulate AprX and LipA production at the transcriptional level (operon *aprX-lipA*) suggesting that the regulation is post-transcriptional or post-translational.

Relating to mineral content, the expression of the *aprX-lipA* operon is negatively regulated at the transcriptional level by iron (III) (Woods et al., 2001). Moreover, McCarthy et al. (2004) observed that the operon *aprX-lipA* is under transcriptional control of the two-component regulatory system homologous to the *E. coli* two-component system called EnvZ-OmpR. However, those authors observed that lipase production was more affected by this regulatory system than peptidase production. The distal locations between the genes *aprX* and *lipA* on the operon could explain that this difference of regulation may be related to their proximal and distal position, respectively, within the *aprX-lipA* operon (McCarthy et al., 2004).

## Control of Spoilage by Heat-Resistant Bacterial Enzymes

Reducing the activity and/or limiting the secretion of heat-resistant hydrolytic enzymes of psychrotrophic bacteria is a scientific challenge. Once the enzymes are formed, reducing their activity by heating seems to be very difficult. A recent review (Stoeckel et al., 2016b) summarizes available data on inactivation of *Pseudomonas* proteases and proposes heat treatments that reduce the protease activity in the final product to entend the shelf life of UHT products. For UHT products intended for export, UHT heating combined with prolonged preheating (e.g., 90–95°C for 180–90 s) is suggested to reduce 99.99% of the indigenous milk plasmin activity. *Pseudomonas* proteases show also an irreversible low temperature inactivation behavior due to unfolding of the tertiary structure of the enzyme at a temperature range of 45–65°C, rendering it susceptible to autoproteolysis. This inactivation was recently shown between 42 and 48°C for a new broad specificity metalloprotease from a *Pseudomonas* spp. isolated from refrigerated milk (Ertan et al., 2015). However, this effect seems much lower in milk compared to buffer systems because of protective effects of milk components, and thus a low preheating step is not effective to sufficiently reduce this bacterial proteolytic activity. The development of other heating processes (e.g., heat-treatment at 125–130°C) for long holding times (>150 s) has been suggested (Stoeckel et al., 2016b). However, such treatment may result in color changes and degradation of lysin, thiamin, and riboflavin (Kessler, 1996).

Another option for spoilage control is to prevent the production of heat-resistant enzymes by limiting the growth of psychrotrophic bacteria in raw milk. This could be realized by modifying the composition of the atmosphere surrounding the milk. N_2_ gas flushing of cold-stored raw milk (6°C) has been shown to strongly inhibit bacterial growth (Gschwendtner et al., 2016), as 3–4 log fewer bacteria were counted after 7 days compared to non-flushed milk. Furthermore, analysis of the bacterial population by next generation sequencing (NGS) of 16S rRNA transcripts showed a relatively lower number of *Pseudomonas* reads in the N_2_ gas flushed milk, indicating selective inhibition of *Pseudomonas* growth. Changing the atmosphere by N_2_ may thus have potential as a control measure to prevent outgrowth of *Pseudomonas* spp. during cold storage of raw milk. However, information in the literature about the ability of facultative anaerobic species of the genus *Bacillus* and its allied genera and of the facultative anaerobic genus *Serratia* to produce extracellular enzymes under anaerobic conditions is lacking at this time. Furthermore, practical applications must be evaluated at dairy farm and industrial level. CO_2_ treatment of raw milk has also been shown to reduce the microbial growth in raw milk (Ma et al., 2003; Vianna et al., 2012; Lo et al., 2016). Lo et al. (2016) observed that in CO_2_-treated raw milk samples a clear inhibition of bacterial growth compared to non-treated samples, resulting in a delay of spoilage by at least seven days. Using NGS, a relatively lower number of *Pseudomonas* and *Serratia* reads were found in three out of five CO_2_-treated raw milk samples, indicating selective growth inhibition of these genera. However, a disadvantage of CO_2_ treatment is that it reduces the pH of the milk (Ma et al., 2003) which may result in changes of (heat)stability.

A third and probably the most cost-effective option for control of spoilage caused by heat-resistant bacterial enzyme activity is hygiene: preventing the contamination of raw milk with psychrotrophic bacteria. Good hygiene practices during milking, cold-storage and transport of the raw milk may reduce the risk of contamination, as these bacteria mainly originate from the udder, milking equipment and milk storage tanks. Wash water used to clean milking equipment has also been shown to be an important source of *Pseudomonas* contamination of raw milk, indicating the need for attention to water quality (Perkins et al., 2009; Leriche and Fayolle, 2012). Specific pre-milking teat cleaning strategies have been shown to reduce the spore count in milk by >1 log (Magnusson et al., 2006). It remains to be investigated whether such udder hygiene management strategies are also effective to reduce the psychrotrophic non-spore forming count in view of the study of Mallet et al. (2012) which showed that teat care has more influence on the composition of technologically relevant microbial groups than on the composition of other groups such as *Pseudomonas* and other Gram-negative bacteria. This may indicate that control of biofilms in milk production and processing environments is maybe more important (reviewed by Marchand et al., 2012; Aswathanarayan and Vittal, 2014; Nucera et al., 2016), as release of vegetative cells or spoilage enzymes from these biofilms may compromise the quality of UHT products (Flach et al., 2014; Teh et al., 2014b). Prevention of biofilm formation may possibly be achieved by specific coating of stainless steel surfaces of milk equipment and milk storing tanks with spoilage bacteria, as was recently shown for milk spore-formers on plate heat exchanger surfaces (Jindal et al., 2016). Biofilms are difficult to remove because the bacteria are protected from disinfectants due to the presence of extracellular polymeric substances. The hygienic design of milking and milk storage equipment as well as effective cleaning and disinfection procedures and proper application are all important factors in the control of biofilms in the dairy industry (Marchand et al., 2012). To remove bacterial biofilms on stainless steel surfaces, the use of specific disinfecting agents, such as products based on hydrogen peroxide and peroxyacetic acid, as well as higher concentrations and longer contact times may be required (Królasik et al., 2010).

Recently, some novel strategies based on the reduction of the bacterial contamination of the raw milk have been proposed as potential measures to extend the shelf life of UHT products, such as the use of microfiltration and the application of lytic bacteriophages. Microfiltration (1.4 μm pore diameter) resulted in 1–2 log reduction of the psychrotrophic bacterial count of the raw milk and ESL by 21 to 63 days of the UHT-treated milk for low and high somatic cell count (SCC) raw milk, respectively (Zhang et al., 2016). The practical application of microfiltration at the farm is questionable, however, since the cream must be removed from the raw milk before such treatment is possible. Application of a lytic phage cocktail against *Pseudomonas* was shown to result in a 1-log reduction of the psychotrophic bacteria of raw milk after 5 days at 4°C (Hu et al., 2016). However, the rather limited effect and the fact that the use of bacteriophages in food is strictly regulated in many countries will probably hamper practical application.

## Conclusion

The combination of psychrotrophic growth in cooled raw milk with the concomitant production of heat-resistant spoilage enzymes presents a formidable challenge to the dairy industry, which relies on refrigerated storage of the raw milk supply and high temperature treatment to produce long shelf life products like UHT-milk and other related dairy products and milk powder. The predominant genus responsible for milk spoilage worldwide found in cold raw milk is *Pseudomonas*, although in specific regions *Serratia* is considered a predominant genus responsible for milk spoilage appearing in cold raw milk. Further research using cultivation-independent metagenomics studies should be performed to exclude possible cultivation biases in most of the studies performed up to now. But bacterial isolates will remain necessary to establish whether they truly produce heat resistant enzymes relevant for spoilage of UHT-milk and related products. The current scientific knowledge on peptidase and lipase enzyme production and activity in these microorganisms still gives no viable possible control options. At present, these microorganisms should be controlled as much as possible at each step of the dairy production chain taking into account an optimal hygiene and cooling management. However, a taxonomically exact and region tailored knowledge of the heat-resistant spoilage enzyme producing microbiota in raw milk will help to trace the contamination sources in the supply and production chain in order to prevent their entrance. The data on the microbiota composition in raw milk presented in this review on a worldwide scale may offer the necessary points of view to look for specific as well as common patterns of contamination with these spoilage microorganisms. On the other hand, a first fast screening at the dairy processing plant of the incoming raw milk for potential heat-resistant spoilage enzymatic activity would be helpful to steer the milk flow toward processing for long shelf life milk products such as UHT-milk or toward other shorter shelf life products. No such test, which would require very high sensitivity, is currently available; its development represents an enormous scientific challenge.

## Author Contributions

SGM, FB, MH, and MV contributed substantially to the conception of this review. SM, JD, and MH have participated in the acquisition of data about the relationship between off-flavors and proteolysis in milk samples. EV contributed to the section on control of spoilage by heat-resistant bacterial enzymes. MV, EV, and JD also provided a critical review of the manuscript.

## Conflict of Interest Statement

The authors declare that the research was conducted in the absence of any commercial or financial relationships that could be construed as a potential conflict of interest.
